# Maternal Methamphetamine Exposure Influences Behavioral Sensitization and Nucleus Accumbens DNA Methylation in Subsequent Generation

**DOI:** 10.3389/fphar.2022.940798

**Published:** 2022-07-19

**Authors:** Nan Dong, Jie Zhu, Rui Wang, Shuai Wang, Yanjiong Chen, Changhe Wang, Eyleen L.K Goh, Teng Chen

**Affiliations:** ^1^ College of Forensic Medicine, Xi’an Jiaotong University Health Science Center, Xi’an, China; ^2^ The Key Laboratory of Health Ministry for Forensic Science, Xi’an Jiaotong University, Xi’an, China; ^3^ Neuroscience Research Center, Institute of Mitochondrial Biology and Medicine, Key Laboratory of Biomedical Information Engineering of the Ministry of Education, School of Life Science and Technology, Xi’an Jiaotong University, Xi’an, China; ^4^ Department of Immunology and Pathogenic Biology, College of Basic Medicine, Xi’an Jiaotong University Health Science Center, Xi’an, China; ^5^ Neuroscience and Mental Health Faculty, Lee Kong Chian School of Medicine, Nanyang Technological University, Singapore, Singapore; ^6^ Singhealth Duke-NUS Neuroscience Academic Clinical Programme, Singapore, Singapore

**Keywords:** methamphetamine, maternal exposure, behavioral sensitization, DNA methylation, nucleus accumbens

## Abstract

The deleterious effects of methamphetamine (METH) exposure extend beyond abusers, and may potentially impact the vulnerability of their offspring in developing addictive behaviors. Epigenetic signatures have been implicated in addiction, yet the characteristics to identify prenatal METH abuse to offspring addiction risk remains elusive. Here, we used escalating doses of METH-exposed mouse model in F0 female mice before and during pregnancy to simulate the human pattern of drug abuse and generated METH-induced behavioral sensitization to investigate the addictive behavior in offspring mice. We then utilized whole genome-bisulfite sequencing (WGBS) to investigate the methylation signature of nucleus accumbens (NAc) in male METH-sensitized mice. Interestingly, male but not female offspring exhibited an enhanced response to METH-induced behavioral sensitization. Additionally, the METH-exposed group of male mice underwent a more comprehensive wave of epigenome remodeling over all genomic elements compared with unexposed groups due to drug exposure history. 104,219 DMCs (METH-SAL vs*.* SAL-SAL) induced by prenatal METH-exposure were positively correlated with that of postnatal METH-exposure (38,570, SAL-METH vs*.* SAL-SAL). Moreover, 4,983 DMCs induced by pre- and postnatal METH exposure (METH-METH vs*.* SAL-METH) were negatively correlated with that of postnatal METH exposure, and 371 commonly changed DMCs between the two comparison groups also showed a significantly negative correlation and 86 annotated genes functionally enriched in the pathways of neurodevelopment and addiction. Key annotated genes included *Kirrel3*, *Lrpprc*, and *Peg3*, implicated in neurodevelopmental processes, were down-regulated in METH-METH group mice compared with the SAL-METH group. Taken together, we render novel insights into the epigenetic correlation of drug exposure and provide evidence for epigenetic characteristics that link maternal METH exposure to the intensity of the same drug-induced behavioral sensitization in adult offspring.

## Introduction

Methamphetamine (METH), the second most commonly used illicit drug in the world (United Nations Office on Drugs and Crime, 2021) behind cannabis, has now become the top threat in China (United Nations Office on Drugs and Crime, 2018). METH is often associated with the experience of increased pleasure during sex, risky sexual behavior, impaired decision-making (Semple et al., 2004), and resulting in unplanned pregnancies (Corsi and Booth, 2008), particularly in teenagers. Epidemiological data show that approximately 1% of deliveries are associated with amphetamine-type stimulants abuse, especially METH, in the united states during 2004–2015 (Wendell, 2013; Ross et al., 2015; Admon et al., 2019). Unlike opioid abusers, which can be treated with methadone replacement, pregnant women with METH dependence have no medication options for METH withdrawal (Karila et al., 2010). Furthermore, METH-related deliveries were associated with worse outcomes and higher costs compared with other deliveries (Gorman et al., 2014; Admon et al., 2019).

Epidemiological data suggest attention-deficit/hyperactivity disorder (ADHD) (LaGasse et al., 2012) and learning and memory deficit in children with prenatal METH experience (Diaz et al., 2014; Ross et al., 2015; Bailey and Diaz-Barbosa, 2018). Limited animal studies on intergenerational transmission show enhanced cocaine-conditioned reward and hyper-locomotion in male offspring mice of METH-exposed parents (Itzhak et al., 2015), whereas impaired recognition memory and spatial memory retention in male offspring mice after maternal METH exposure (Dong et al., 2018). However, it remains elusive to determine the impact of prenatal METH abuse on offspring addiction risk. Therefore, the key challenge for research is to ascertain the factors that drive individual susceptibility to substance abuse and to provide effective prevention strategies.

Epigenetic mechanisms enable individuals to adapt to the dynamic changes in surroundings, such as nutrition, stress, and drug abuse (Feng and Nestler, 2013; Cadet et al., 2016). DNA methylation is one of the known epigenetic modifications, which cannot only respond to environmental effects (Feng and Nestler, 2013) but also can be stably inherited across generations (Vassoler et al., 2013; Watson et al., 2015). Thus, genome-wide approach to DNA methylation sequencing has immense potential to identify novel molecular targets and pathways most closely linked to the intergenerational effects of METH exposure (Hansen et al., 2012; Wang et al., 2013; Cavalli and Heard, 2019).

The nucleus accumbens (NAc) is considered to be a key nucleus of the mesolimbic system involved in regulating drug addiction (Scofield et al., 2016). The NAc mainly receives dopaminergic projections from the ventral tegmental area (VTA) (Beckstead et al., 1979), and glutamatergic projections from the prefrontal cortex (Ma et al., 2014), ventral CA1 region of the hippocampus (Zhou et al., 2019), basolateral amygdala (Lee et al., 2013), and paraventricular thalamus (Hsu et al., 2014). Meanwhile, the NAc also projects GABAergic back into VTA (Yang et al., 2018). Thus, the critical integration of dopaminergic and glutamatergic signaling in the NAc was thought to be the neuronal basis of addictive behaviors (Gardner, 2011; Scofield et al., 2016). In addition, previous studies of us found that NAc play a critical role in the long-term maintenance and expression of METH-induced sensitization (Su et al., 2019; Wang et al., 2022). Besides, we also found that the expression patterns of neurodevelopment-related genes in the NAc of F1 offspring mice were altered after prenatal METH exposure (Dong et al., 2018). However, DNA methylation profiling of NAc to determine the risk of prenatal METH abuse for addictive behavior in offspring is not well understood.

We hypothesized that epigenetic alterations involving DNA methylation are associated with the effects of maternal METH exposure on offspring, and these changes affect gene expression with relevance to behavioral consequences. To test this, we established an intermittent and gradually escalating METH regime in F0 female mice to mimic the drug abuse pattern of female teenagers, which usually begins before pregnancy and tends to continue the use even during gestation to avoid negative symptoms after withdrawal (Admon et al., 2019), and then evaluated the vulnerability to drug addictive behavior by METH-induced behavioral sensitization model in adult F1 offspring mice. Then, we performed genome-wide DNA methylation analysis by whole-genome bisulfite sequencing (WGBS) in METH-sensitized mice NAc and detected the mRNA expression of differential methylated regions (DMRs) annotated genes by quantitative PCR. Hence, we supplied a resource pool for further functional study of epigenetic modifications of specific genes in mediating the effects of METH-induced behavioral sensitization of offspring.

## Materials and Methods

### Drugs

Methamphetamine (METH) hydrochloride was purchased from the National Institute for Control of Pharmaceutical and Biological Products (Beijing, China) and dissolved in 0.9% NaCl (saline). All the drugs and saline were administered intraperitoneally (*i.p.*) in a volume of 10 ml kg^−1^ body weight except the dams during the gestation period (subcutaneously, *s. c.*).

### Animals

Adult C57BL/6 mice (8 weeks old, male and female, 20–25 g) were purchased from the Beijing Vital River Laboratory Animal Technology Co., Ltd. (Beijing, China). All mice were housed under a 12-h light/dark cycle (lights on from 7:00 a.m. to 7:00 p.m.) and were housed in groups of four with food and water provided *ad libitum*. Both temperature (23 ± 1°C) and humidity (50 ± 5%) of the housing room were controlled. All animal protocols used in our current study were approved by the Institutional Animal Care and Use Committee of Xi’an Jiaotong University. All efforts were made to reduce the number of animals used and minimize their suffering. All the mice experienced 7 days of habituation, during which each mouse was handled for 2 min once daily before initiating behavior testing. Male and female offspring mice were used for METH-induced sensitization as described below, with at least eight litters and a maximum of two pups from the same litter representing each group.

### Intergenerational Methamphetamine Animal Model

Adult C57BL/6 mice were mated to produce at least 20 male and 42 female offspring that were born on the same day. Following the weaning process on postnatal day (PD) 28, four mice (F0, the first generation) of the same sex were housed in the same cage. Female mice at PD 32 were randomly assigned to two treatment groups: METH (*n* = 22) and saline (*n* = 20). Starting from PD 33, females received every-other-day injections (*i.p.*) of saline or an escalating dose of METH as follows: PD 33–39, 0.5 mg kg^−1^; PD 40–46, 1 mg kg^−1^; PD 47–53, 2 mg kg^−1^; PD 54–60, 4 mg kg^−1^. On PD 60, saline and METH-treated females were mated with untreated males. From PD 61 onwards, saline or a fixed dose of METH −4 mg·kg^−1^ was subcutaneously (s.c.) injected every-other-day into saline or METH-treated females respectively. The gestational length was 19 days for both groups, so all injections were terminated on gestation day 17. This method of drug exposure mimics human drug abuse-escalating doses starting in adolescence and continuing during pregnancy did not produce dopaminergic neurotoxicity (Itzhak et al., 2015; Dong et al., 2018).

Our previous study has reported that treatment of F0 dams with METH reduced the rate of pregnancy by about 20% without observed effects on other maternal behaviors, including pregnancy complications, weight change during lactation, the length of gestation, and the total number of pups per litter. In addition, there were also no differences in litter characteristics, such as the number of alive pups, male/female ratio in the litter, and body weight gain of pups from PD3-PD27 (Dong et al., 2018).

**FIGURE 1:**
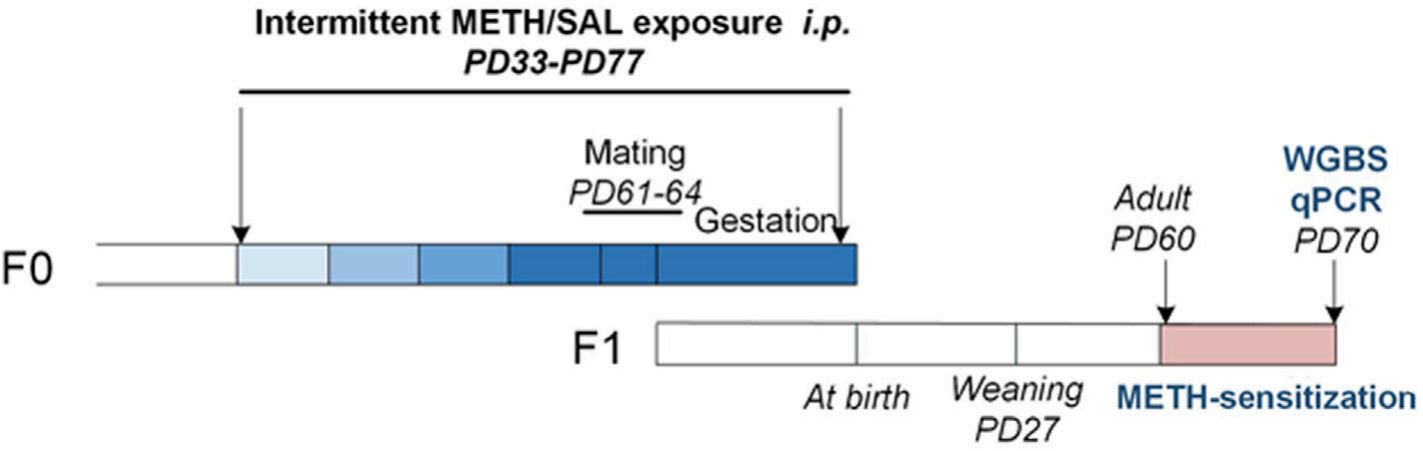
Experimental procedure for female F0 mice METH treatment and F1 offspring mice along with approximate age ranges. The color lump from light to dark blue represents escalating doses of METH (0.5/1/2/4 mg kg^−1^). Abbreviations: F0, F1 (two generations of age matched mouse families), PD, postnatal day; WGBS, whole genome bisulfite sequencing; qPCR, quantitative polymerase chain reaction.

Subsequently, F1 from the same litter were randomly divided into the saline and METH groups (both sexes) for subsequent METH-induced behavioral sensitization to avoid the litter effect: saline pups (males, *n* = 16 and females, *n* = 16) and METH pups (males, *n* = 16 and females, *n* = 16) (Figure 1).

### Methamphetamine-Induced Behavioral Sensitization

**FIGURE 2:**
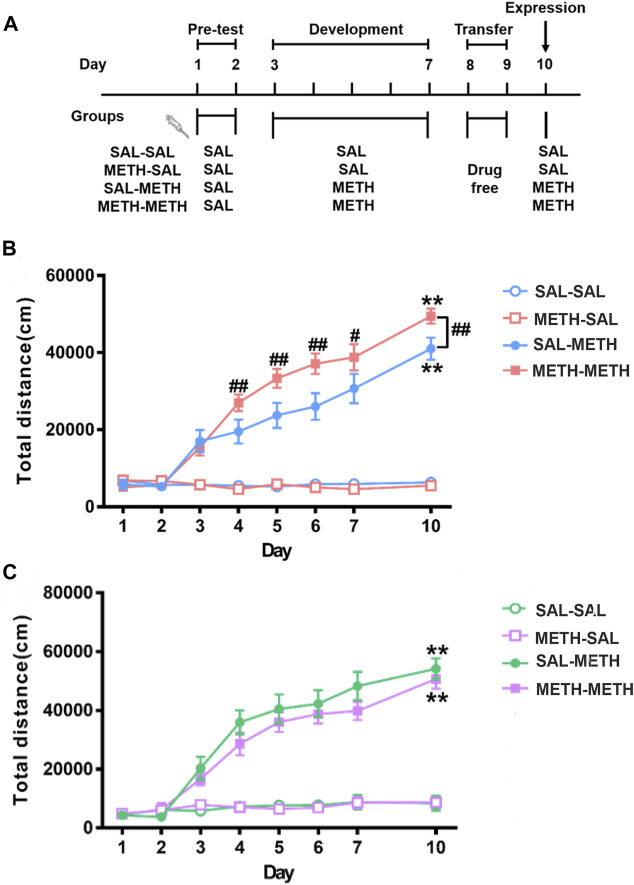
Maternal METH exposure enhanced METH-induced behavioral sensitization in F1 adult male progeny. **(A)** Schematics of METH-induced locomotor sensitization. **(B)** METH- METH male mice developed an earlier locomotor sensitization and exhibited hyper-locomotor activity compared with SAL-METH males. **(C)** METH-METH female mice developed a trend of reduced behavioral sensitization compared with SAL-METH females. Two-way repeated measures ANOVA with a post hoc multiple comparisons was used. **p < 0.01 compared with day 3; #*p* < 0.05, ##*p* < 0.01 compared with SAL-METH group. Data are presented as mean ± SEM (each group *n* = 8). Abbreviations: SAL-SAL, prenatal and postnatal saline exposure; METH-SAL, prenatal METH and postnatal saline exposure; SAL-METH, prenatal saline and postnatal METH exposure; METH-METH, prenatal and postnatal METH exposure.

The METH injection paradigm used in this study has been previously demonstrated to produce robust locomotor sensitization (Zhao, 2014). F1 offspring (PD 61) were given once-daily injections of saline for two consecutive days (days 1–2), after which they were randomly grouped into four. The groups of mice then received once-daily intraperitoneal (*i.p.*) injections of METH (SAL-METH and METH-METH group, 1 mg kg^−1^, *n* = 8 each group) or saline (SAL-SAL and METH-SAL group, *n* = 8 each group) for five consecutive days (day 3–7). After five injected days, the mice were housed in cages for two injection-free days (day 8–9). On day 10, the mice received a challenging injection of either METH (1 mg kg^−1^) or saline (Figure 2A). Before the beginning of experiments, mice were habituated to the experiment room for 30 min, then allowed to freely explore the test chamber (43 cm × 43 cm × 43 cm high) and their locomotor activities were recorded by a Smart Video Tracking System (Version 2.5, Panlab Technology for Bioresearch, Spain) for 1 h after the injections.

### NAc Extraction and Preparation

After chronic METH-induced behavioral sensitization, F1 males from the four groups SAL-SAL, METH-SAL, SAL-METH, and METH-METH (Figure 2A) were sacrificed by cervical dislocation and the NAc (+1.70 mm from Bregma) was removed and immediately frozen in liquid nitrogen. Genomic DNA was extracted using a TIANamp Genomic DNA Kit (TIANGEN Biotech, China) according to the manufacturer’s instructions and then preserved in TE buffer and ∼250 ng genomic DNA was used for WGBS. Meanwhile, the total RNA of NAc was extracted by ^TM^RNA Isolation Kit-Tissue (EXIQON, United States) following the procedures and then preserved in Elution buffer.

### Whole Genome Bisulfite Sequencing

Library preparation was performed by Annoroad Genomics. A260/280 ratio (1.8–2.0) and agarose gel electrophoresis were used to determine the quality and integrity of DNA samples. For library preparation, ∼250 ng gDNA was fragmented to ∼300 bp, then end-repaired, dA-tailed and ligated to methylated Illumina adapters following the user manual of Truseq DNA Sample Prep Kit (Illumina, United States), The 250–350 bp fragments were selected by 2% agarose gel, and subjected to bisulfite conversion by EZ DNA Methylation-Gold™ Kit (D5006, Zymo, United States), and PCR amplified by ZymoTaq PreMix (Zymo, United States). The libraries were sequenced PE150 on Illumina Hiseq 4,000. Three biological replicates were sequenced for each group. Raw sequencing data that support the findings of this study have been deposited in the Sequence Read Archive with accession code PRJNA844300 and the access URL (https://dataview.ncbi. nlm. nih.gov/object/PRJNA844300?reviewer = brli4l54mj01dj1bdoujl2jah6).

### Sequencing Data Analysis

The raw WGBS sequencing reads were quality checked and trimmed for low quality (Phred quality score <30) nucleotides, adapters, and artificially introduced bases in the end-repair step. In the 12 samples sequenced, the average clean Q30 bases rate (error rate less than 0.1%) was above 95%, and high reproducibility in biological replicates. Alignment and methylation calling of the unique mapped reads were then performed with Bismark (Krueger and Andrews, 2011) using genome assembly for *Mus musculus* Ensembl (GRCm38.83, RefSeq gene annotations). Differential methylation regions (DMRs) were defined as regions of the genome containing at least three CpGs and >10 × depth of coverage, and we required the presence of a minimum of one statistically significant CpGs (FDR <0.01) with a concordant (either hypo- or hyper-methylated) mean methylation difference 20% between prenatal or postnatal METH and corresponding control groups, DMRs were considered significant with *p* < 0.05 (Li et al., 2013).

Averaged methylation level profiles of all differentially methylated CpG sites (DMCs) from 5 Kb upstream of transcription start sites (TSS), through scaled gene bodies (exon and intron) to 5 Kb downstream of transcription termination sites (TTS) (Akalin et al., 2012). The Z-score normalization of methylation level in heatmap was clustered based on Euclidean distances with R package “pheatmap”. Venn diagram and correlation analysis of DMCs affected between different comparison groups were based on the Pearson correlation coefficient. Functional enrichment of commonly methylated DMCs localized genes was mapped to cellular component GO terms in the Ontology database and KEGG pathway-related database (Kanehisa et al., 2017). We illustrated DNA methylation changes of DMR localized genes using integrative genomics viewer and expanded views of DNA methylation patterns for gene loci using the UCSC genome browser. DMRs were considered differentially methylated if their FDR was at most 0.05.

### Statistical Analysis

We first analyzed the behavioral data in F1 METH-induced behavioral sensitization with repeated measures factorial design (pre-exposure factor*post-exposure factor) analysis of variance (ANOVA), and then we re-combined the two levels (saline and METH) of the two factors (pre- and post-exposure) due to the interaction between the two factors and re-analyzed with repeated measures ANOVA followed by Bonferroni post hoc tests. Real-time qPCR data were analyzed by one-way ANOVA by Bonferroni post hoc tests. Exploratory behavior in the open field and elevated plus-maze was analyzed by Student’s t test. For statistical analysis of WGBS, data were presented in the section on sequencing data analysis. *p* < 0.05 was considered statistically significant. Data are presented as mean ± SEM.

## Results

### Enhanced Methamphetamine-Induced Sensitization in Adult Male F1 Offspring

To explore the potential intergenerational effects of maternal METH exposure, we examined METH (1 mg kg^−1^, *i. p.*) induced behavioral sensitization in adult F1 offspring. The repeated measures factorial design (pre-exposure factor*post-exposure factor) ANOVA was first used and revealed that significant main effects of pre-exposure in males (males: F _(1,27)_ = 6.238, *p* = 0.019; females: F _(1,26)_ = 1.700, *p* = 0.204); and post-exposure in males and females (males: F _(1,27)_ = 248.445, *p* < 0.01; females: F _(1,26)_ = 122.009, *p* < 0.01) as well as their interactions in males (males: F _(1,27)_ = 7.528, *p* = 0.01; females: F _(1,26)_ = 1.677, *p* = 0.207). Then, we re-combined the two levels (saline and METH) of the two factors (pre- and post-exposure) due to the interaction between the two factors, and re-analyzed with repeated measures ANOVA followed by Bonferroni post hoc tests.

As shown in Figure 2B and Figure 2C, repeated measures of ANOVA revealed significant main effects of day (males: F _(7, 21)_ = 62.480, *p* < 0.001; females: F _(7, 20)_ = 32.338, *p* < 0.001), group (males: F _(3, 27)_ = 82.908, *p* < 0.001; females: F _(3, 26)_ = 40.796, *p* < 0.001) as well as their interactions (males: F _(21, 60)_ = 3.358, *p* < 0.001; females: F _(21, 66)_ = 1.924, *p* < 0.023). In order to analyze the differences between different time points within group, we adjusted the degrees of freedom of time-related F values in the repeated measures ANOVA test. Both male and female METH-METH and SAL-METH group mice developed significant locomotor sensitization (males: day 10 vs*.* day 3, *p* < 0.001; females: day 10 vs*.* day 3, *p* < 0.001). Multiple comparisons among different time points within each group found that METH-METH males (vs*.* SAL-METH males) developed an earlier locomotor sensitization (METH-METH: day 4 vs*.* day 3, *p* < 0.001; SAL-METH: day 4 vs*.* day 3, *p* = 0.427; day 5 vs*.* day 3, *p* < 0.01). Multiple comparison among different groups at each time point found that METH-METH males (vs*.* SAL-METH males) exhibited hyper-locomotor activity (day 4, *p* < 0.001; day 5, *p* < 0.01; day 6. *p* < 0.001; day 7, *p* < 0.05; day 10, *p* < 0.01). Taken together, the abnormal behaviors observed in F1 male offspring clearly demonstrate intergenerational behavioral disturbances in association with maternal history of METH exposure.

### Dynamics of Genome-wide DNA Methylation Profiles Following Prenatal or Postnatal Methamphetamine Exposure

**FIGURE 3:**
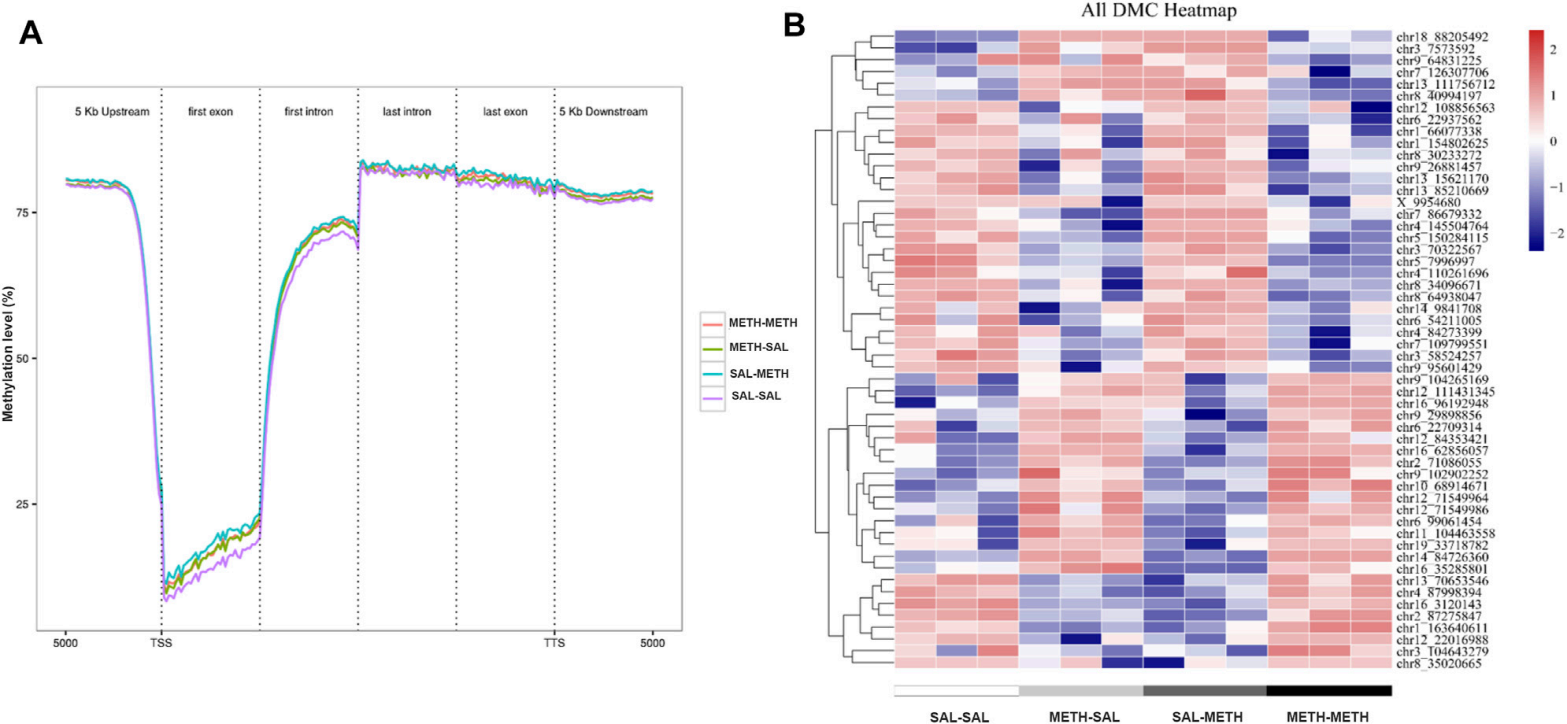
DNA methylation dynamics in NAc of mice prenatal or postnatal METH exposure conditions. **(A)** Averaged methylation level profiles of all differentially methylated CpG sites (DMCs) from 5 Kb upstream of transcription start sites (TSS), through scaled gene bodies (exon and intron) to 5 Kb downstream of transcription termination sites (TTS). **(B)** Hierarchical clustering of differentially methylated CpG in NAc of prenatal or postnatal saline/METH exposure mice relative to corresponding control mice. Hyper- and hypomethylated CpG are represented in red and blue color, respectively. Data in each group are based on *n* = 3.

To determine the role of DNA methylation in the regulation of behavioral responses following prenatal or postnatal METH exposure, we then sought to identify genome-wide DNA methylation changes in the adult NAc. Using the behaviorally characterized male cohort of prenatal or postnatal METH- or saline-treated mice, we performed whole-genome bisulfite sequencing (WGBS) to assess DNA methylation sites in NAc samples from SAL-SAL, METH-SAL, SAL-METH, and METH-METH group mice. In the 12 samples sequenced, an average clean Q30 bases rate (error rate less than 0.1%) was above 95%, and high reproducibility in biological replicates was demonstrated. So, we combined the biological replicates in each group for subsequent analysis. We first calculated the association of generation (F0, F1) and phenotype (METH, saline) traits with the global methylation profiles. METH-exposed groups underwent a more comprehensive wave of epigenome remodeling over all genomic elements compared with unexposed groups in CpG motif, likely due to the effect of METH intake; also there was a difference between SAL-METH and METH-METH groups in transcription start sites (TSS) and first exon, indicating possible association of DNA methylation with re-exposure to METH (Figure 3A). Besides, we calculated the methylation level and frequency of the different motifs of mC (mCG, mCHG, and mCHG, H represents the other three non-G bases, H = A/C/T), and found that the methylation ratio of CpG motif was the highest in the three motifs in all four groups, while the methylation ratio of the three motifs varied in different groups. The frequency of methylation of CHG and CHH motifs was generally lower than that of CpG motif (data not shown). Therefore, we mainly focused on the methylation changes of CpG motif in our in-depth analysis.

Subsequently, we performed regional analysis to detect significant methylation differences in 1,000 base-pair sliding regions (*q* ≤ 0.05) and detected differences at single CpG resolution (*q* ≤ 0.05) by focusing on those significant 200 base-pair regions. A total of 1,570 sliding regions were differentially methylated (DMRs) in the METH-SAL group compared with the SAL-SAL group, 710 DMRs were found in SAL-METH vs*.* SAL-SAL comparison group, 1,060 DMRs in METH-METH vs*.* METH-SAL comparison group, and 562 DMRs in METH-METH vs*.* SAL-METH comparison group (Table 1). Within these regions, 104,219, 38,570, 31,528, and 4,983 single CpG sites were differentially (*q* ≤ 0.05) methylated (DMCs) in SAL-SAL vs*.* METH-SAL comparison group, SAL-SAL vs*.* SAL-METH comparison group, METH-SAL vs*.* METH-METH comparison group and SAL-METH vs*.* METH-METH comparison group, respectively. These DMCs clustering results were consistent with the behavioral sensitization profile (Figure 3B), suggesting that dynamic changes in METH-induced DMCs could be the imprints of the prenatal or postnatal drug experience. DNA methylation dynamics among the four groups: among the 1,570 differentially methylated sites present in METH-SAL vs*.* SAL-SAL comparison group, 58.9% were hyper-methylated and 40.1% were hypo-methylated (Table 1). Also, a similar global methylation profile was found in METH-METH vs*.* SAL-METH comparison group: 57.1 and 42.9% of the 562 DMRs were hyper- and hypo-methylated, respectively (Table 1).

**TABLE 1:** Genomic distribution of DMR measured by WGBS in adult offspring prenatally or postnatally exposed to METH compared to corresponding control group.

Genomics Regions	Number of Regions	Total Regions (%)	Hypermethylated (%)	Hypomethylated (%)
SAL_SAL-vs-METH_SAL^a^				
DMR	1,570	100	925 (58.9172)	645 (41.0828)
Exons	71	4.52	34 (47.8873)	37 (52.1127)
Intergenic	756	48.15	440 (58.2011)	316 (41.7989)
Introns	700	44.59	432 (61.7143)	268 (38.2857)
Promoter	43	2.74	19 (44.1860)	24 (55.8140)
CpG islands	17	1.08	16 (94.1176)	1 (5.8824)
SAL_SAL-vs-SAL_METH^b^				
DMR	710	100	565 (79.5775)	145 (20.4225)
Exons	43	6.06	34 (79.0698)	9 (20.9302)
Intergenic	365	51.41	277 (75.8904)	88 (24.1096)
Introns	285	40.14	238 (83.5088)	47 (16.4912)
Promoter	17	2.39	16 (94.1176)	1 (5.8824)
CpG islands	16	2.25	16 (100.000)	0 (0.000)
METH_SAL-vs-METH_METH^c^				
DMR	1,060	100	719 (67.8302)	341 (32.1698)
Exons	70	6.60	49 (70.0000)	21 (30.0000)
Intergenic	507	47.83	333 (65.6805)	174 (34.3195)
Introns	444	41.89	310 (69.8198)	134 (30.1802)
Promoter	39	3.68	27 (69.2308)	12 (30.7692)
CpG islands	15	1.42	12 (80.0000)	3 (20.00)
SAL_METH-vs-METH_METH^d^				
DMR	562	100	321 (57.1174)	241 (42.8826)
Exons	41	7.30	21 (51.2195)	20 (48.7805)
Intergenic	255	45.37	152 (59.6078)	103 (40.3922)
Introns	240	42.70	133 (55.4167)	107 (44.5833)
Promoter	26	4.63	15 (57.6923)	11 (42.3077)
CpG islands	7	1.25	4 (57.1429)	3 (42.8571)

a Mice prenatally METH-exposed compared with prenatally saline-exposed.

b Mice postnatally METH-exposed compared with postnatal saline-exposed.

c Mice prenatally and postnatally METH-exposed compared with prenatally METH-exposed.

d Mice prenatally and postnatally METH-exposed compared with postnatal METH-exposed.

### Positive Correlation of DMCs Between Prenatal and Postnatal Methamphetamine Exposure

**FIGURE 4:**
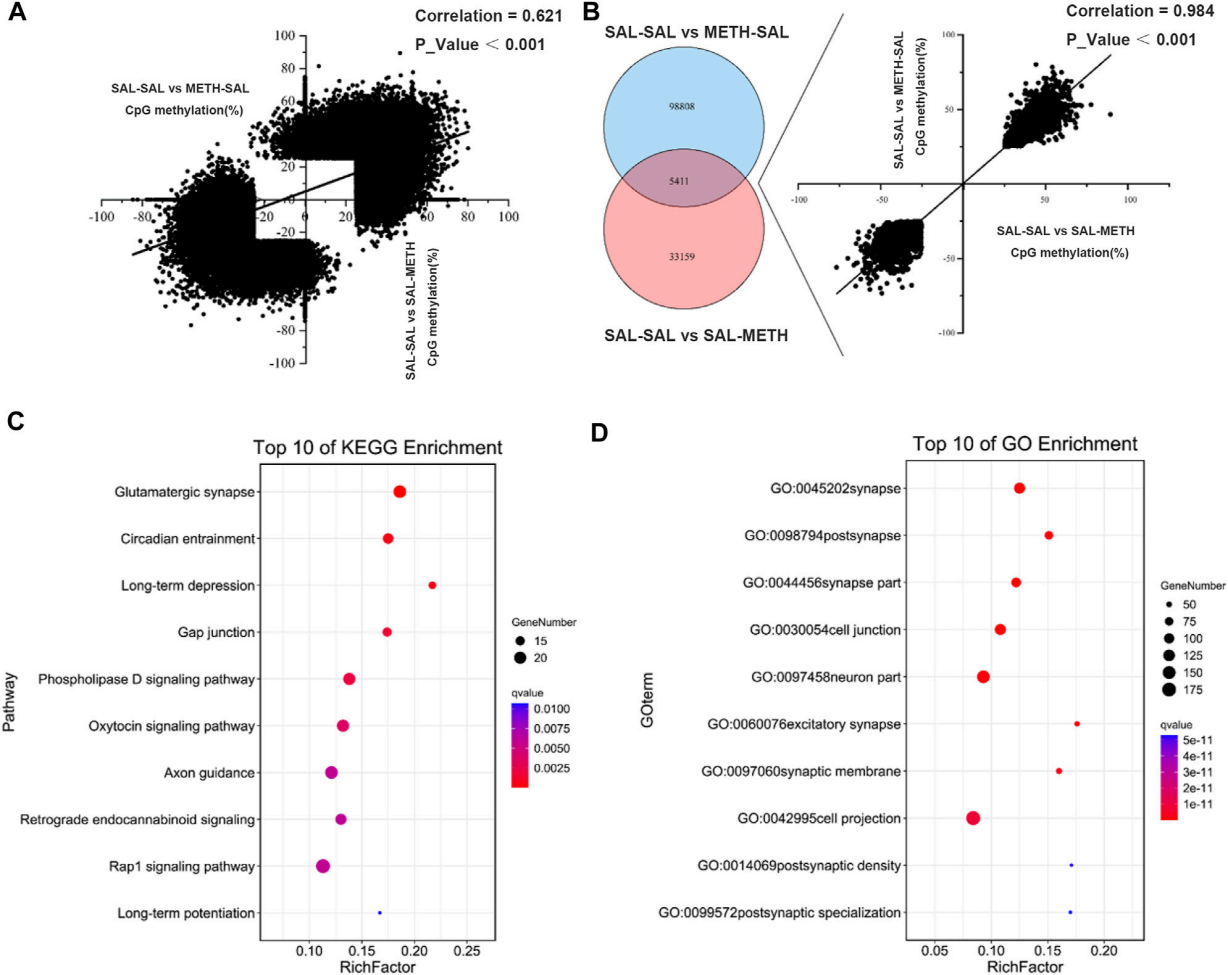
Venn diagram depicting the number of CpGs that are uniquely and commonly affected between prenatal METH exposure and postnatal METH exposure comparison group. **(A)** A positive correlation of DMCs between prenatal METH exposure and postnatal METH exposure comparison group. **(B)** 5,411 commonly methylated CpGs (co-methylated) affected by prenatal METH exposure and postnatal METH treatment and show a significant positive correlation. **(C)** Top 10 pathway enrichment results of co-methylated CpG localized genes. **(D)** Top 10 cellular components of GO enrichment results of co-methylated CpG localized genes. Hypergeometric distribution test was used. Data in each group are based on *n* = 3.

There was a positive correlation (r = 0.6206, *p <* 0.001) among all CpG sites that were differentially methylated in postnatal METH treatment (SAL-SAL vs*.* SAL-METH; 38,570 sites) or prenatal METH treatment (SAL-SAL vs*.* METH-SAL; 104,219 sites) mice (Figure 4A). 5,411 CpG sites were commonly affected by pre- and postnatal METH treatments, as confirmed by the hypergeometric distribution test (Figure 4B). The direction of methylation changes in these common CpG sites correlated between prenatal and postnatal exposure conditions (r = 0.984, *p <* 0.001): those sites that were hyper-methylated (or hypo-methylated) in prenatal METH treatment mice were also hyper-methylated (or hypo-methylated) in postnatal METH treatment mice. These co-methylated CpG annotated to 1,055 unique genes were subjected to gene ontology (GO) term and KEGG pathway enrichment analysis, to explore whether the co-methylated genes in two conditions shared the specific functional features. For both prenatal and postnatal treatment conditions, the most significant enrichment occurred in GO term category cellular component involved genes annotating with the term synapse (Figure 4C); and the most significant enrichment in the KEGG pathway was glutamatergic synapse, followed by circadian entrainment and long-term depression (Figure 4D).

### Negative Correlation of DMCs Between Postnatal and Pre- and Postnatal Methamphetamine Exposure

**FIGURE 5:**
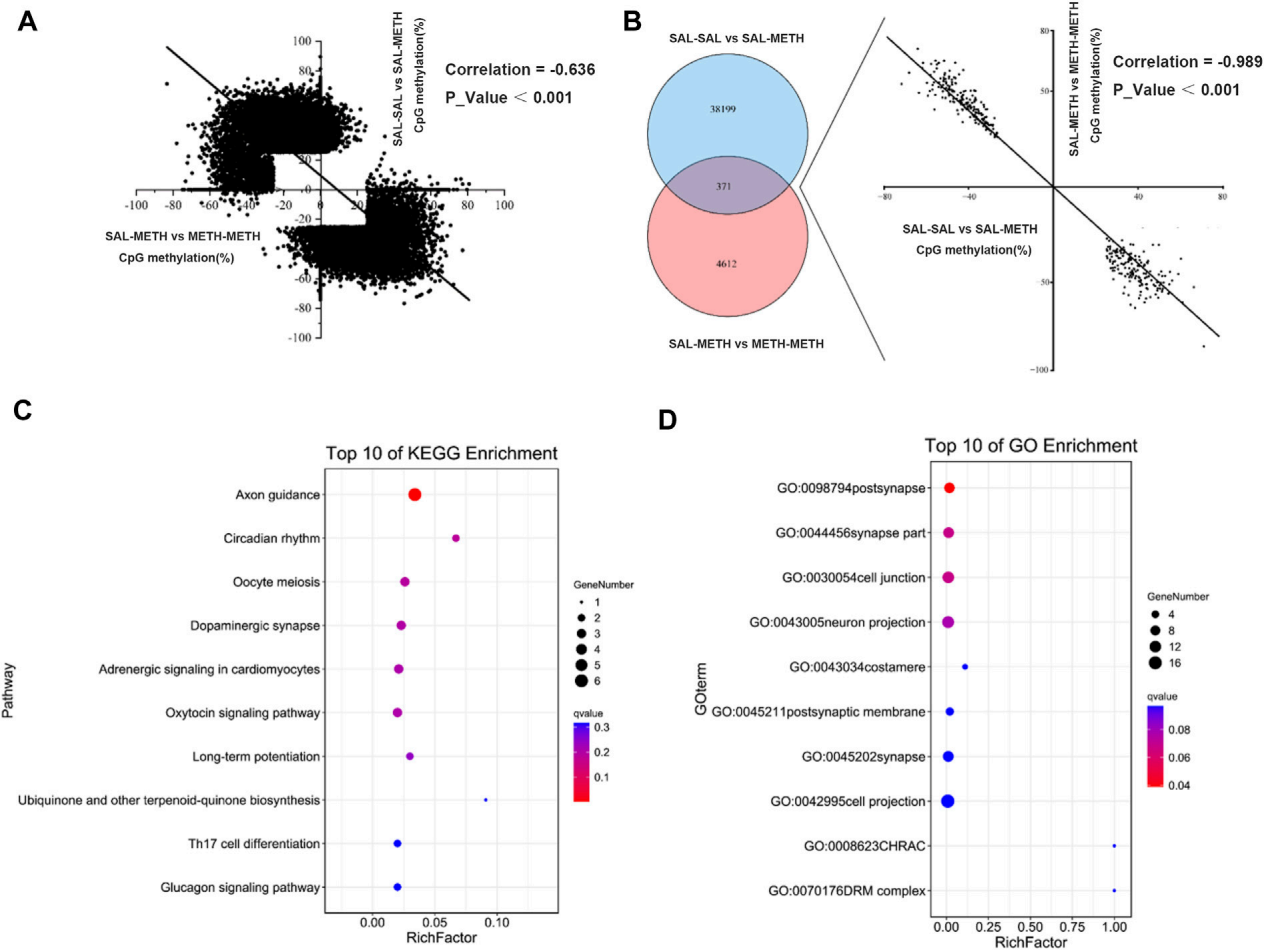
Venn diagram depicting the number of CpGs that are uniquely and commonly affected between postnatal METH exposure and pre- and postnatal METH exposure comparison group. **(A)** A negative correlation of DMCs between postnatal METH exposure and pre- and postnatal METH exposure comparison group. **(B)** 371 commonly methylated CpGs (co-methylated) affected by postnatal and pre- and postnatal METH treatments and show a significant negative correlation. **(C)** Top 10 pathway enrichment results of co-methylated CpG localized genes. **(D)** Top 10 cellular components of GO enrichment results of co-methylated CpG localized genes. Hypergeometric distribution test was used. Data in each group are based on *n* = 3.

To reveal the mechanistic characteristics of prenatal METH-exposed offspring that exhibited more sensitized to METH, we also analyzed the correlation relationship between postnatal METH group (SAL-SAL vs*.* SAL-METH) and pre- and postnatal METH group (SAL-METH vs*.* METH-METH). Surprisingly, DMCs affected by postnatal METH treatment negatively correlated to DMCs affected by pre- and postnatal METH treatment (r = −0.636, *p <* 0.001; Figure 5A). However, 371 CpGs were commonly affected by postnatal and pre- and postnatal METH treatments, and the direction of methylation changes in these common CpG sites correlated between postnatal and pre- and postnatal exposure conditions (r = −0.989, *p <* 0.01): those sites that were hyper-methylated (or hypo-methylated) in postnatal METH treatment mice were hypo-methylated (or hyper-methylated) in pre- and postnatal METH treatment mice (Figure 5B). These co-methylated CpG annotated to 86 unique genes, that were enriched into GO term and KEGG pathway. For both postnatal and pre- and postnatal treatment conditions, the most significant enrichment occurred in GO term category cellular component involving genes annotated with the term post-synapse (Figure 5C); and the most significant enrichment in the KEGG pathway was axon guidance, followed by circadian rhythm, oocyte meiosis and dopaminergic synapse (Figure 5D), which may attribute to enhanced sensitization in pre- and postnatal METH group.

### Altered mRNA Expression of Co-methylated Genes Following Prenatal or Postnatal Methamphetamine Exposure

**FIGURE 6:**
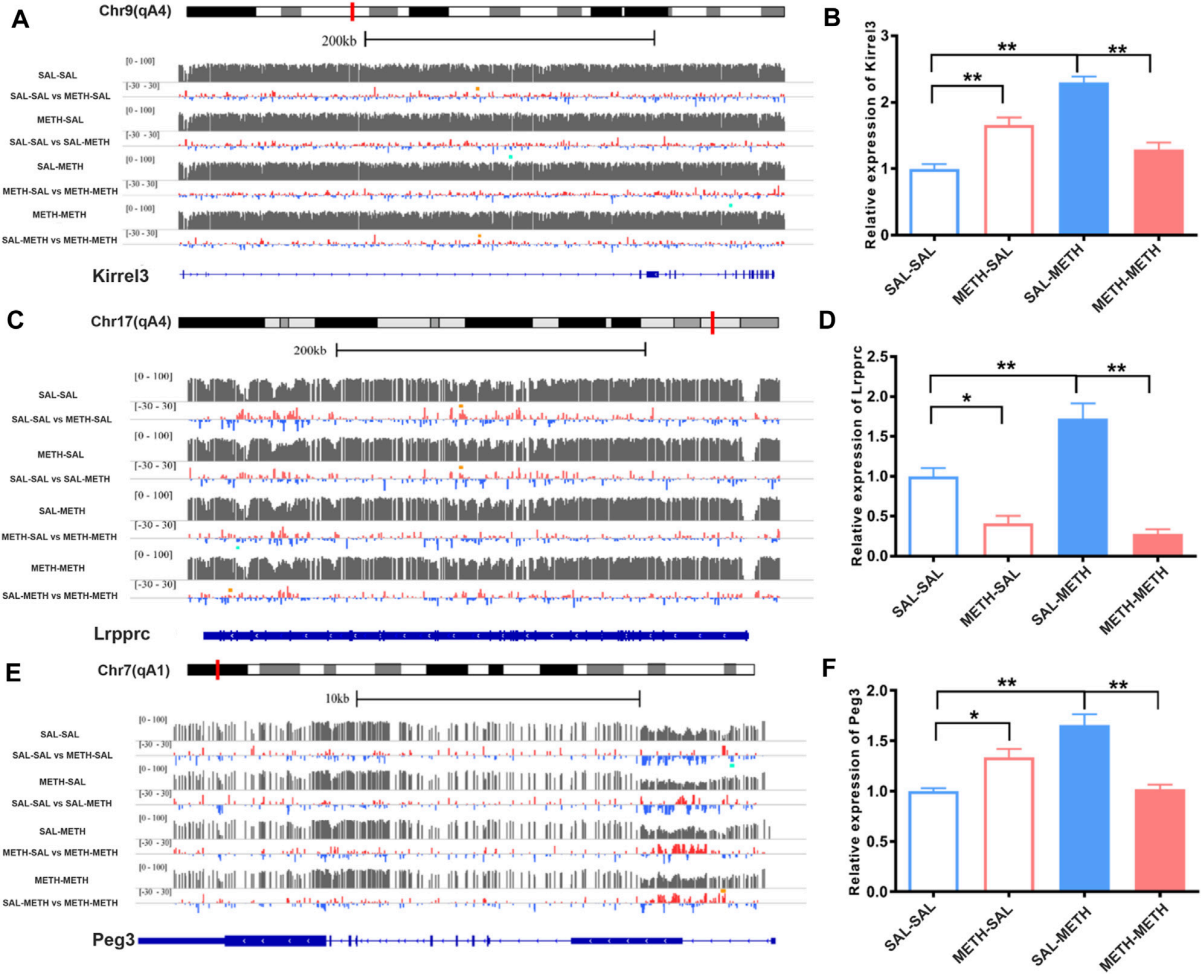
Illustration of DNA methylation changes of Kirrel3, Lrpprc, and Peg3 genes in NAc of prenatal or postnatal METH-exposed males. Left panel **(A,C,E)** shows expanded views of DNA methylation patterns for sample gene loci, visualized using the UCSC genome browser: Kirrel3; Lrpprc; and Peg3. For each gene, tracks 1, 3, 5, and 7 show the average methylation intensity of the saline group, prenatal METH group (METH-SAL), postnatal METH group (SAL-METH), and pre- and postnatal METH group (METH- METH) respectively. Tracks 2, 4, 6, and 8 show average methylation differences between the two experimental conditions. Green dots represent regions significantly hypo-methylated corresponding to the correspondence control track. Orange dots represent regions significantly hyper-methylated corresponding to the correspondence control track. DMRs were considered differentially methylated if their FDR was at most 0.05. The last track shows exons and introns based on the mouse NCBI RNA reference sequences collection (RefSeq). Right panel **(B,D,F)** shows changes in expression of Kirrel3, Lrpprc, and Peg3 genes in NAc of prenatal or postnatal METH -exposed males. one-way ANOVA followed by a Bonferroni post hoc test was used. *p < 0.05,**p < 0.01 compared with the corresponding control mice, respectively. SAL-SAL, pre- and postnatal SAL exposure; METH-SAL, prenatal METH and postnatal SAL exposure; SAL-METH, prenatal SAL, and postnatal METH exposure; METH-METH, pre- and postnatal METH exposure. Data in each group are based on *n* = 3. qPCR data are presented as Mean ± SEM.

We found two genes with differential methylation modification changes in the two comparison groups: *Kirrel3* (kin of IRRE-like protein 3) and *Lrpprc* (Leucine-rich PPR-motif-containing protein). As shown in Figure 6 A, hyper-methylated of *Kirrel3* in SAL-SAL vs*.* METH-SAL comparison group and SAL-METH vs*.* METH-METH comparison group (orange dot), and hypo-methylated *Kirrel3* in SAL-SAL vs*.* SAL-METH comparison group (blue dot) were detected. In Figure 6B, up-regulation of *Kirrel3* mRNA expression was observed in METH-SAL group and SAL-METH group compared with the corresponding control groups, and down-regulated of *Kirrel3* in METH-METH compared with SAL-METH. Hyper-methylated *Kirrel3* in SAL-SAL vs*.* METH-SAL, SAL-SAL vs*.* SAL-METH, and SAL-METH vs*.* METH-METH comparison group (orange dot) were shown in Figure 6C,D, down-regulated *Lrpprc* mRNA expression was observed in METH-SAL group and METH-METH group compared with the corresponding control groups, while there was an up-regulated *Lrpprc* expression in SAL-METH compared with SAL-SAL group. Next, we examined the methylation level of *Peg3* (paternally expressed 3)—a transcription factor. *Peg3* was hypo-methylated in SAL-SAL vs*.* METH-SAL comparison group and hyper-methylated in SAL-METH and METH-METH comparison group. Similarly, qPCR results show up-regulated *Peg3* in SAL-METH compared with SAL-SAL group, but down-regulated in METH-METH group compared with SAL-METH group (Figures 6E,F). These down-regulated DMRs localized genes may attribute to enhanced sensitization in pre- and postnatal METH group.

## Discussion

Methamphetamine (METH) is one of the most accessible drugs in China and worldwide (United Nations Office on Drugs and Crime, 2021). Women abusing recreational drugs before pregnancy tend to continue the use even during gestation to avoid negative symptoms after withdrawal (Finnegan, 2013; Admon et al., 2019). Therefore, children born to mothers, who abused METH during pregnancy, have an increased risk of substance abuse after they grow up is of critical importance. The METH exposure regimen used in our study mimics the human drug addicts' experience that starts usually in adolescence and continues through adulthood, and then through pregnancy (initiation, escalation of use, addiction) (Itzhak et al., 2015; Dong et al., 2018).

Behavioral or psychomotor sensitization, as a progressive and enduring response, can be observed upon being challenged with the same or lower dose of a drug after withdrawal of repeated drug exposure (Mead et al., 2004). The present data show that METH-METH males exhibit enhanced METH-induced locomotor activity and developed an earlier locomotor sensitization (the second METH challenge) compared with SAL-METH males (Figure 2B), indicating prenatal METH exposure indeed increased vulnerability to METH-induced sensitization in adult male progeny. Since METH can pass into the fetus from the placental barrier during prenatal exposure (Ross et al., 2015), it is assumed that F1 offspring mice experienced prolonged withdrawal between prenatal and postnatal METH exposure. Therefore, our results also imply that once enhanced drug sensitivity develops, it can persist for extended periods of time. Meanwhile, we found no significant drug sensitivity differences between METH-METH females and SAL-METH females (Figure 2C). Consistent with previous studies, the female gender appears to be a protective factor (Jacobsen et al., 2007; Etaee et al., 2017; Traccis et al., 2020). In the current study, we paid more attention to the cause of the enhanced sensitization effects of male offspring, further studies are needed to explore the differential effects between male and female mice.

Genetic and environmental risks for substance use disorders (addictive behavior) typically do not only add together but also interact with each other over development. DNA methylation modification enables more flexible regulatory mechanisms and diversity (Eckersley-Maslin et al., 2018; Skvortsova et al., 2018), while the dynamic changes of methylation modification allow better environmental adaptability in response to stimuli (Schmitt et al., 2014; Ficz, 2015). Nucleus accumbens (NAc) are known to receive both dopaminergic inputs from ventral tegmental area (VTA) and glutamate inputs from the medial prefrontal cortex (mPFC), and play a critical role in the long-term maintenance and expression of METH-induced behavioral sensitization (Scofield et al., 2016). Alterations in adult NAc methylation may serve as a legacy of prenatal METH exposures, which may contribute to the intergenerational transmission of METH-induced sensitized phenotype. Our results show that prenatal METH exposure affects the comprehensive level of epigenome remodeling of overall genomic elements (Figure 3A). And the differential methylated CpG sites (DMCs) clustering in different groups were consistent with METH exposure groupings, indicating that dynamic changes of CpG methylation sites could be imprints of prenatal or postnatal METH experiences (Figure 3B).

Our correlation analysis of DMCs shows a positive correlation between prenatal METH treatment and postnatal METH treatment, suggesting a similar direction of methylation changes between prenatal and postnatal exposure conditions. Also, the common methylated DMCs were positively correlated, and prenatal and postnatal METH exposure alter common pathways such as glutamatergic synapse, circadian entrainment, long-term depression, gap junction and phospholipase D signaling pathway (top 5), which have been reported in association with METH addiction (Pendergast et al., 2012; Jayanthi et al., 2014; Cadet et al., 2015; Castellano et al., 2016; Etaee et al., 2017; Limanaqi et al., 2018). Meanwhile, we also found the DMCs between prenatal METH exposure and prenatal and postnatal METH exposure were negatively correlated, indicating these negatively correlated DMCs may underlie the neural basis of the enhanced sensitization effects in prenatal and postnatal METH males. We further analyzed common DMCs annotated genes and found that they were enriched in pathways such as axon guidance, circadian rhythm, oocyte meiosis, dopaminergic synapse, and adrenergic signaling in cardiomyocytes (top 5). Interestingly, we found that the gene *Ppp2r2b* (protein phosphatase two regulatory subunit B, beta isoform) was a conserved serine/threonine protein phosphatase involved in the dopaminergic pathway, which has been found implicated in neurological disorders (Rampino et al., 2019; Wang et al., 2019), as well as METH-induced behavioral sensitization in our previous report (Chen et al., 2020). This suggests that methylation changes of neurodevelopmental processes-related genes may contribute to enhanced sensitization effects in prenatal and postnatal METH-exposed males. Besides, METH can alter fetal development through a wide variety of mechanisms. For example, METH crosses the placenta to act on the developing fetal brain directly, but can also exert effects through a variety of organs within the mother, including the uterus, placenta, heart, lungs, and brain (Jones and Fielder, 2015; Ross et al., 2015). Consistent with our pathway enrichment results of negatively correlated DMCs between postnatal and prenatal and postnatal METH exposure, oocyte meiosis and adrenergic signaling in cardiomyocyte pathways were affected. As the main focus here is on the intergenerational transmission of DNA methylation, whether it can be transmitted to F2 offspring has not been thoroughly investigated.

The phenotypic response to prenatal METH exposure resulted from the comprehensive effect of a large set of dysregulated genes. Among the vast DMRs localized genes, we found that two genes-*Kirrel3* and *Lrpprc* have been annotated in all four comparison groups. *Kirrel3* encodes an immune adhesion molecular involved in synaptic formation, synaptic transmission, and ultrastructure. In humans, loss of the KIRREL3 gene was associated with neurodevelopmental disorders, such as Jacobson’s syndrome, intellectual disability, and autism spectrum disorder (Bhalla et al., 2008). Knockout of the *Kirrel3* gene in mice led to auditory sensory and motor skill impairment and ADHD (attention deficit and hyperactivity disorder) (Hisaoka et al., 2018), as well as an increased risk of ADHD in children following prenatal METH exposure (Pendergast et al., 2012; Petzold et al., 2021). *Lrpprc* is a leucine-rich PPR motif-containing protein, and mutation of the LRPPRC gene may lead to Leigh syndrome in French Canadians (Mootha et al., 2003). A recent study reported that the LRPPRC gene was DMRs annotated in alcohol use disorder subjects (Gatta et al., 2021), one of the adverse implications of prenatal METH exposure, such as polysubstance abuse (Ross et al., 2015; Li et al., 2021). Besides, we found down-regulation of differentially methylated genes (*Kirrel3*, *Lrpprc*, *Peg3*) in METH-METH males compared to SAL-METH males, but up-regulated in METH-METH females (not shown). Differential expression of these genes may contribute to sexually dimorphic sensitization effects after prenatal and postnatal METH exposure. While this study focuses on the increased vulnerability of prenatal and postnatal METH-induced behavioral sensitization in male progeny, it will be of interest to further explore the underlying mechanism of METH-induced behavioral sensitization between male and female progeny in the near future.

## Conclusion

Our study provides evidence that maternal METH exposure increased the vulnerability to the same drug-METH in male progeny. We also found dynamic DNA methylation changes in the NAc, including DMRs, localized to genes with important roles in neurodevelopmental processes and addiction, that may serve as a predictor of subsequent addiction risk in male progeny. These findings, along with the growing use of METH among young people who may subsequently bear children (United Nations Office on Drugs and Crime, 2018), highlight the importance of further investigations into the long-term effects of drug exposure not only during the individual’s lifetime but also on future generations.

## Data Availability

The datasets presented in this study can be found in online repositories. The names of the repository/repositories and accession number(s) can be found below: https://www.ncbi.nlm.nih.gov/, PRJNA844300.

## Abbreviation

F0, F1, two generations of age-matched mouse families; PD, postnatal day; WGBS, whole-genome bisulfite sequencing; qPCR, quantitative polymerase chain reaction; NAc, nucleus accumbens.
